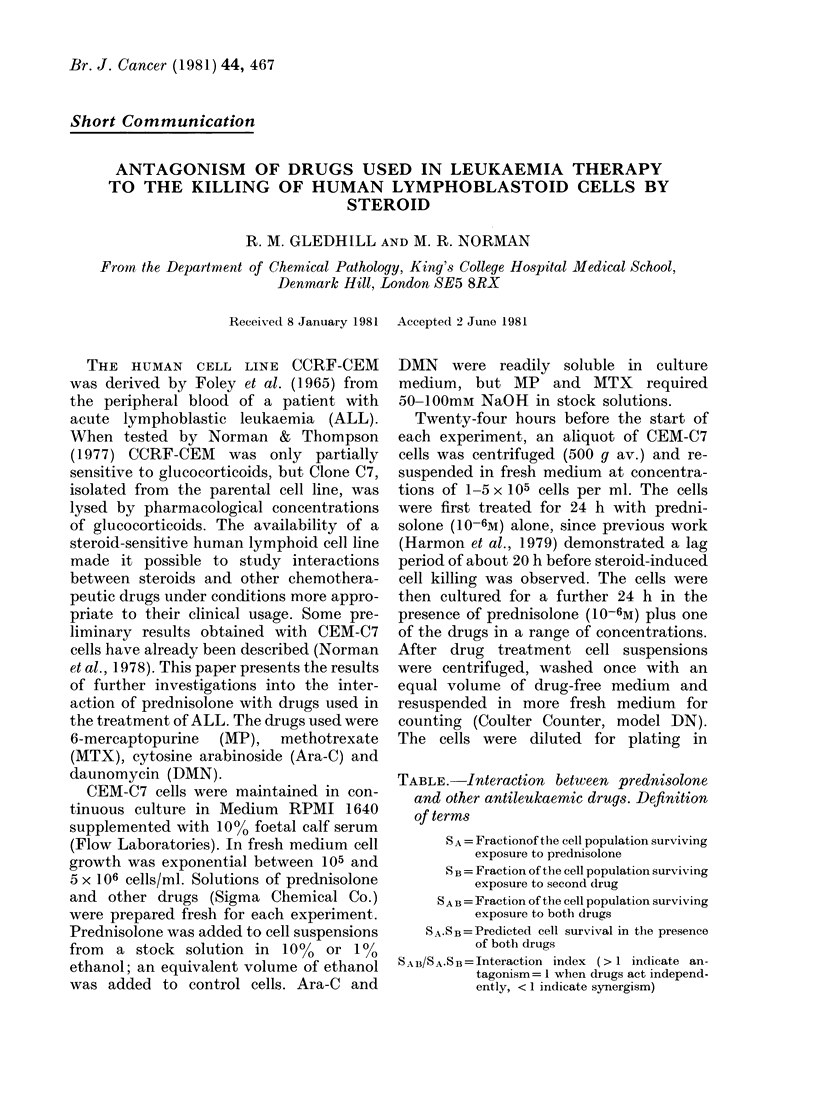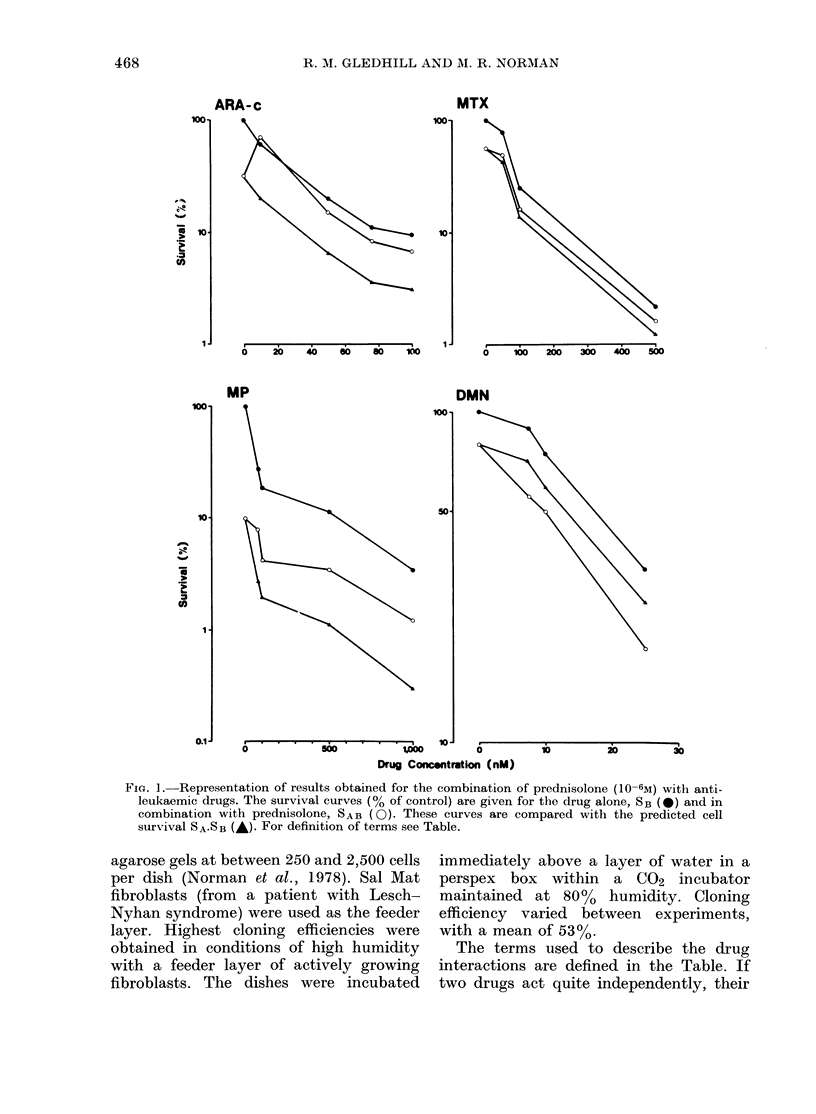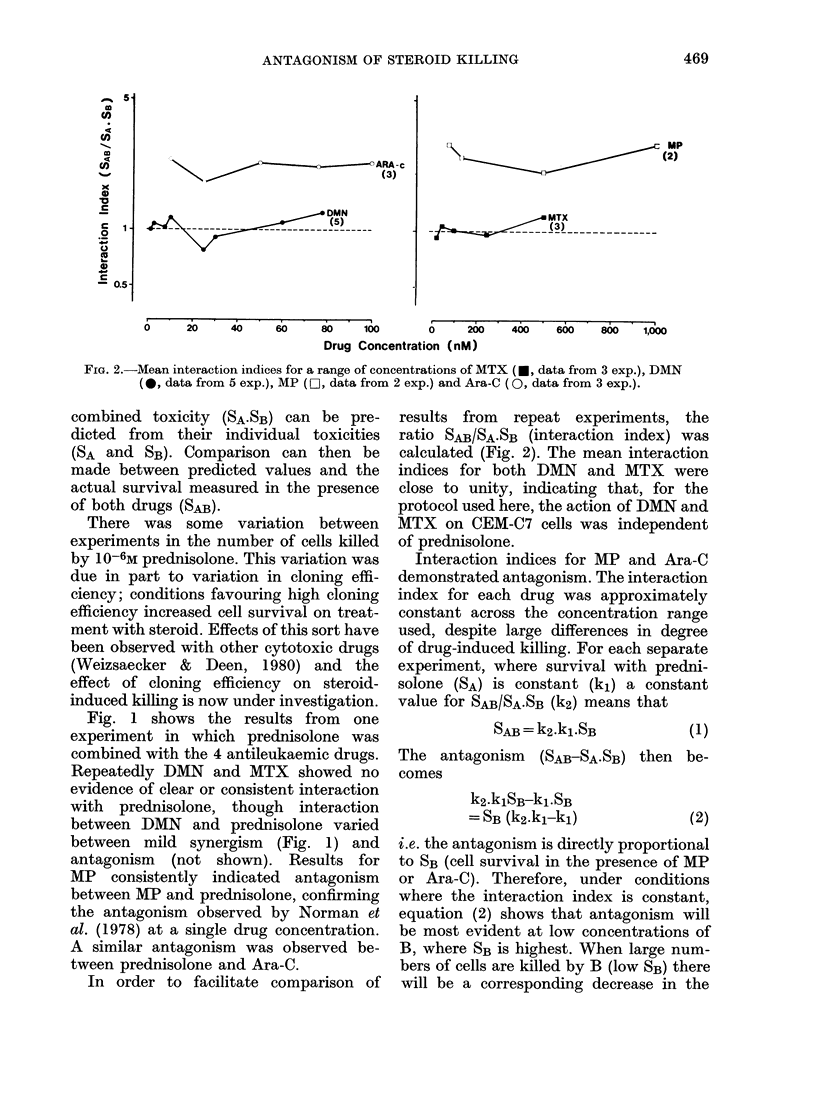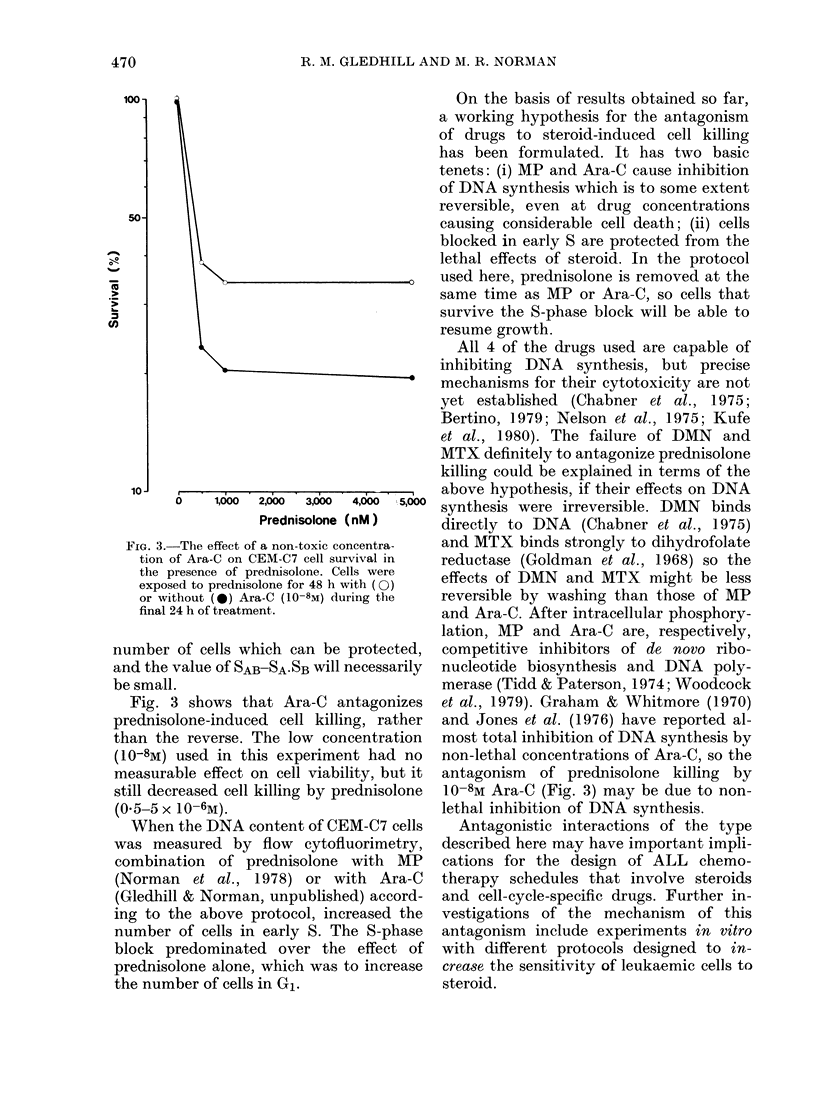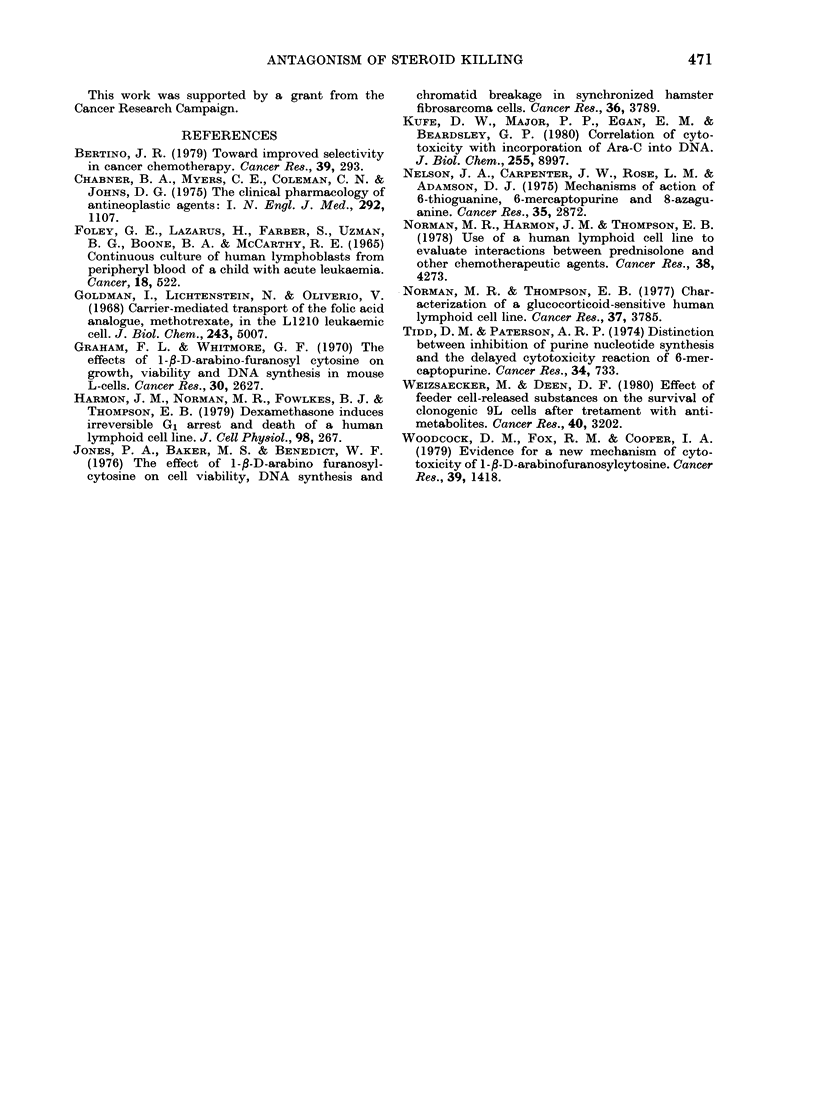# Antagonism of drugs used in leukaemia therapy to the killing of human lymphoblastoid cells by steroid.

**DOI:** 10.1038/bjc.1981.208

**Published:** 1981-09

**Authors:** R. M. Gledhill, M. R. Norman


*Br. J. Cancer* (1981) **44**, 467

***Short Communication***

### ANTAGONISM OF DRUGS USED IN LEUKAEMIA THERAPY TO THE KILLING OF HUMAN LYMPHOBLASTOID CELLS BY STEROID

R. M. GLEDHILL AND M. R. NORMAN

*From the Department of Chemical Pathology, King's College Hospital Medical School, Denmark Hill, London SE5 8RX*

Received 8 January 1981 Accepted 2 June 1981

THE HUMAN CELL LINE CCRF-CEM was derived by Foley *et al.* (1965) from the peripheral blood of a patient with acute lymphoblastic leukaemia (ALL). When tested by Norman & Thompson (1977) CCRF-CEM was only partially sensitive to glucocorticoids, but Clone C7, isolated from the parental cell line, was lysed by pharmacological concentrations of glucocorticoids. The availability of a steroid-sensitive human lymphoid cell line made it possible to study interactions between steroids and other chemotherapeutic drugs under conditions more appropriate to their clinical usage. Some preliminary results obtained with CEM-C7 cells have already been described (Norman *et al.*, 1978). This paper presents the results of further investigations into the interaction of prednisolone with drugs used in the treatment of ALL. The drugs used were 6-mercaptopurine (MP), methotrexate (MTX), cytosine arabinoside (Ara-C) and daunomycin (DMN).

CEM-C7 cells were maintained in continuous culture in Medium RPMI 1640 supplemented with 10% foetal calf serum (Flow Laboratories). In fresh medium cell growth was exponential between $10^5$ and $5 \times 10^6$ cells/ml. Solutions of prednisolone and other drugs (Sigma Chemical Co.) were prepared fresh for each experiment. Prednisolone was added to cell suspensions from a stock solution in 10% or 1% ethanol; an equivalent volume of ethanol was added to control cells. Ara-C and DMN were readily soluble in culture medium, but MP and MTX required 50–100mM NaOH in stock solutions.

Twenty-four hours before the start of each experiment, an aliquot of CEM-C7 cells was centrifuged (500 *g* av.) and resuspended in fresh medium at concentrations of $1–5 \times 10^5$ cells per ml. The cells were first treated for 24 h with prednisolone ($10^{-6}$M) alone, since previous work (Harmon *et al.*, 1979) demonstrated a lag period of about 20 h before steroid-induced cell killing was observed. The cells were then cultured for a further 24 h in the presence of prednisolone ($10^{-6}$M) plus one of the drugs in a range of concentrations. After drug treatment cell suspensions were centrifuged, washed once with an equal volume of drug-free medium and resuspended in more fresh medium for counting (Coulter Counter, model DN). The cells were diluted for plating in

TABLE.—*Interaction between prednisolone and other antileukaemic drugs. Definition of terms*

| | |
|---|---|
| $S_A$ = | Fraction of the cell population surviving exposure to prednisolone |
| $S_B$ = | Fraction of the cell population surviving exposure to second drug |
| $S_{AB}$ = | Fraction of the cell population surviving exposure to both drugs |
| $S_A.S_B$ = | Predicted cell survival in the presence of both drugs |
| $S_{AB}/S_A.S_B$ = | Interaction index (> 1 indicate antagonism = 1 when drugs act independently, < 1 indicate synergism) |

FIG. 1.—Representation of results obtained for the combination of prednisolone ($10^{-6}$M) with anti-leukaemic drugs. The survival curves (% of control) are given for the drug alone, $S_B$ (●) and in combination with prednisolone, $S_{AB}$ (○). These curves are compared with the predicted cell survival $S_A.S_B$ (▲). For definition of terms see Table.

agarose gels at between 250 and 2,500 cells per dish (Norman *et al.*, 1978). Sal Mat fibroblasts (from a patient with Lesch–Nyhan syndrome) were used as the feeder layer. Highest cloning efficiencies were obtained in conditions of high humidity with a feeder layer of actively growing fibroblasts. The dishes were incubated immediately above a layer of water in a perspex box within a $CO_2$ incubator maintained at 80% humidity. Cloning efficiency varied between experiments, with a mean of 53%.

The terms used to describe the drug interactions are defined in the Table. If two drugs act quite independently, their

FIG. 2.—Mean interaction indices for a range of concentrations of MTX (■, data from 3 exp.), DMN (●, data from 5 exp.), MP (□, data from 2 exp.) and Ara-C (○, data from 3 exp.).

combined toxicity ($S_A.S_B$) can be predicted from their individual toxicities ($S_A$ and $S_B$). Comparison can then be made between predicted values and the actual survival measured in the presence of both drugs ($S_{AB}$).

There was some variation between experiments in the number of cells killed by $10^{-6}$M prednisolone. This variation was due in part to variation in cloning efficiency; conditions favouring high cloning efficiency increased cell survival on treatment with steroid. Effects of this sort have been observed with other cytotoxic drugs (Weizsaecker & Deen, 1980) and the effect of cloning efficiency on steroid-induced killing is now under investigation.

Fig. 1 shows the results from one experiment in which prednisolone was combined with the 4 antileukaemic drugs. Repeatedly DMN and MTX showed no evidence of clear or consistent interaction with prednisolone, though interaction between DMN and prednisolone varied between mild synergism (Fig. 1) and antagonism (not shown). Results for MP consistently indicated antagonism between MP and prednisolone, confirming the antagonism observed by Norman *et al.* (1978) at a single drug concentration. A similar antagonism was observed between prednisolone and Ara-C.

In order to facilitate comparison of results from repeat experiments, the ratio $S_{AB}/S_A.S_B$ (interaction index) was calculated (Fig. 2). The mean interaction indices for both DMN and MTX were close to unity, indicating that, for the protocol used here, the action of DMN and MTX on CEM-C7 cells was independent of prednisolone.

Interaction indices for MP and Ara-C demonstrated antagonism. The interaction index for each drug was approximately constant across the concentration range used, despite large differences in degree of drug-induced killing. For each separate experiment, where survival with prednisolone ($S_A$) is constant ($k_1$) a constant value for $S_{AB}/S_A.S_B$ ($k_2$) means that

$$S_{AB} = k_2.k_1.S_B \qquad (1)$$

The antagonism ($S_{AB}$–$S_A.S_B$) then becomes

$$k_2.k_1S_B - k_1.S_B = S_B\,(k_2.k_1 - k_1) \qquad (2)$$

*i.e.* the antagonism is directly proportional to $S_B$ (cell survival in the presence of MP or Ara-C). Therefore, under conditions where the interaction index is constant, equation (2) shows that antagonism will be most evident at low concentrations of B, where $S_B$ is highest. When large numbers of cells are killed by B (low $S_B$) there will be a corresponding decrease in the

FIG. 3.—The effect of a non-toxic concentration of Ara-C on CEM-C7 cell survival in the presence of prednisolone. Cells were exposed to prednisolone for 48 h with (○) or without (●) Ara-C ($10^{-8}$M) during the final 24 h of treatment.

number of cells which can be protected, and the value of $S_{AB}$–$S_A.S_B$ will necessarily be small.

Fig. 3 shows that Ara-C antagonizes prednisolone-induced cell killing, rather than the reverse. The low concentration ($10^{-8}$M) used in this experiment had no measurable effect on cell viability, but it still decreased cell killing by prednisolone ($0{\cdot}5$–$5 \times 10^{-6}$M).

When the DNA content of CEM-C7 cells was measured by flow cytofluorimetry, combination of prednisolone with MP (Norman *et al.*, 1978) or with Ara-C (Gledhill & Norman, unpublished) according to the above protocol, increased the number of cells in early S. The S-phase block predominated over the effect of prednisolone alone, which was to increase the number of cells in $G_1$.

On the basis of results obtained so far, a working hypothesis for the antagonism of drugs to steroid-induced cell killing has been formulated. It has two basic tenets: (i) MP and Ara-C cause inhibition of DNA synthesis which is to some extent reversible, even at drug concentrations causing considerable cell death; (ii) cells blocked in early S are protected from the lethal effects of steroid. In the protocol used here, prednisolone is removed at the same time as MP or Ara-C, so cells that survive the S-phase block will be able to resume growth.

All 4 of the drugs used are capable of inhibiting DNA synthesis, but precise mechanisms for their cytotoxicity are not yet established (Chabner *et al.*, 1975; Bertino, 1979; Nelson *et al.*, 1975; Kufe *et al.*, 1980). The failure of DMN and MTX definitely to antagonize prednisolone killing could be explained in terms of the above hypothesis, if their effects on DNA synthesis were irreversible. DMN binds directly to DNA (Chabner *et al.*, 1975) and MTX binds strongly to dihydrofolate reductase (Goldman *et al.*, 1968) so the effects of DMN and MTX might be less reversible by washing than those of MP and Ara-C. After intracellular phosphorylation, MP and Ara-C are, respectively, competitive inhibitors of *de novo* ribonucleotide biosynthesis and DNA polymerase (Tidd & Paterson, 1974; Woodcock *et al.*, 1979). Graham & Whitmore (1970) and Jones *et al.* (1976) have reported almost total inhibition of DNA synthesis by non-lethal concentrations of Ara-C, so the antagonism of prednisolone killing by $10^{-8}$M Ara-C (Fig. 3) may be due to non-lethal inhibition of DNA synthesis.

Antagonistic interactions of the type described here may have important implications for the design of ALL chemotherapy schedules that involve steroids and cell-cycle-specific drugs. Further investigations of the mechanism of this antagonism include experiments *in vitro* with different protocols designed to *increase* the sensitivity of leukaemic cells to steroid.

This work was supported by a grant from the Cancer Research Campaign.

### REFERENCES

BERTINO, J. R. (1979) Toward improved selectivity in cancer chemotherapy. *Cancer Res.*, **39**, 293.

CHABNER, B. A., MYERS, C. E., COLEMAN, C. N. & JOHNS, D. G. (1975) The clinical pharmacology of antineoplastic agents: I. *N. Engl. J. Med.*, **292**, 1107.

FOLEY, G. E., LAZARUS, H., FARBER, S., UZMAN, B. G., BOONE, B. A. & MCCARTHY, R. E. (1965) Continuous culture of human lymphoblasts from peripheryl blood of a child with acute leukaemia. *Cancer*, **18**, 522.

GOLDMAN, I., LICHTENSTEIN, N. & OLIVERIO, V. (1968) Carrier-mediated transport of the folic acid analogue, methotrexate, in the L1210 leukaemic cell. *J. Biol. Chem.*, **243**, 5007.

GRAHAM, F. L. & WHITMORE, G. F. (1970) The effects of 1-$\beta$-D-arabino-furanosyl cytosine on growth, viability and DNA synthesis in mouse L-cells. *Cancer Res.*, **30**, 2627.

HARMON, J. M., NORMAN, M. R., FOWLKES, B. J. & THOMPSON, E. B. (1979) Dexamethasone induces irreversible $G_1$ arrest and death of a human lymphoid cell line. *J. Cell Physiol.*, **98**, 267.

JONES, P. A., BAKER, M. S. & BENEDICT, W. F. (1976) The effect of 1-$\beta$-D-arabino furanosylcytosine on cell viability, DNA synthesis and chromatid breakage in synchronized hamster fibrosarcoma cells. *Cancer Res.*, **36**, 3789.

KUFE, D. W., MAJOR, P. P., EGAN, E. M. & BEARDSLEY, G. P. (1980) Correlation of cytotoxicity with incorporation of Ara-C into DNA. *J. Biol. Chem.*, **255**, 8997.

NELSON, J. A., CARPENTER, J. W., ROSE, L. M. & ADAMSON, D. J. (1975) Mechanisms of action of 6-thioguanine, 6-mercaptopurine and 8-azaguanine. *Cancer Res.*, **35**, 2872.

NORMAN, M. R., HARMON, J. M. & THOMPSON, E. B. (1978) Use of a human lymphoid cell line to evaluate interactions between prednisolone and other chemotherapeutic agents. *Cancer Res.*, **38**, 4273.

NORMAN, M. R. & THOMPSON, E. B. (1977) Characterization of a glucocorticoid-sensitive human lymphoid cell line. *Cancer Res.*, **37**, 3785.

TIDD, D. M. & PATERSON, A. R. P. (1974) Distinction between inhibition of purine nucleotide synthesis and the delayed cytotoxicity reaction of 6-mercaptopurine. *Cancer Res.*, **34**, 733.

WEIZSAECKER, M. & DEEN, D. F. (1980) Effect of feeder cell-released substances on the survival of clonogenic 9L cells after tretament with antimetabolites. *Cancer Res.*, **40**, 3202.

WOODCOCK, D. M., FOX, R. M. & COOPER, I. A. (1979) Evidence for a new mechanism of cytotoxicity of 1-$\beta$-D-arabinofuranosylcytosine. *Cancer Res.*, **39**, 1418.